# AMPK activation, eEF2 inactivation, and reduced protein synthesis in the cerebral cortex of hibernating chipmunks

**DOI:** 10.1038/s41598-019-48172-7

**Published:** 2019-08-15

**Authors:** Shintaro Yamada, Taito Kamata, Hiroyuki Nawa, Tsuneo Sekijima, Nobuyuki Takei

**Affiliations:** 1 Department of Environmental Science and Technology, Graduate School of Science and Technology, Niigata University, Niigata, 951-8585 Japan; 2 Department of Molecular Neurobiology, Brain Research Institute, Niigata University, Niigata, 951-8585 Japan

**Keywords:** Neurochemistry, Molecular neuroscience, Animal physiology

## Abstract

During hibernation, mammalian cells are exposed to severe environmental stressors such as low temperature, lowered O_2_ supply, and glucose deficiency. The cellular metabolic rate is markedly reduced for adapting to these conditions. AMP-activated protein kinase (AMPK) senses the cellular energy status and regulates metabolism. Therefore, we examined AMPK signaling in several brain regions and peripheral tissues in hibernating chipmunk. Eukaryotic elongation factor 2 (eEF2) is a downstream target of AMPK. Phosphorylation of eEF2, indicating its inactivation, is enhanced in the cerebral cortex of hibernating chipmunks. The study indicated that the sequential regulation of AMPK-mammalian target of rapamycin complex 1-eEF2 signaling was altered and protein synthesis ability was reduced in the cerebral cortex of hibernating chipmunks.

## Introduction

Hibernation is an adaptive strategy of some mammals to survive the severe cold and food scarcity in winter. It is a drastic circannual phenotypic alteration in such mammals. In chipmunks, the body temperature falls to nearly 0 °C and heartbeat reduces dramatically during hibernation^1^. In general, the cells of hibernators can tolerate low temperature and low O_2_ supply as well as energy deficiency, presumably reducing the metabolic rate of glucose, lipids, and proteins.

It has been proposed that the circannual hibernation of chipmunks is regulated by hibernating proteins (HPs) produced in the liver and worked in the brain during hibernation^1,2^, which suggests that hibernation is triggered in the brain. It is widely accepted that hibernation is associated with downregulation of metabolism and ensuing energy insufficiency, at least in the brain, although it is unclear whether these metabolic changes are the cause or the result of hibernation^3,4^. Therefore, this study aimed to analyze and compare the energy-sensing and anabolic signaling in the brains, and peripheral tissues of hibernating chipmunks.

AMP-activated protein kinase (AMPK), a heterotrimeric serine/threonine protein kinase, comprising a catalytic α subunit and regulatory β and γ subunits, senses the cellular energy status by detecting the AMP/ATP ratio and regulates metabolism^5^. AMPK is activated when ATP levels fall in situation such as nutrient insufficiency and hypoxia. AMPK activity is coupled with phosphorylation of Thr172 residue of AMPKα subunit^5–7^. When activated by reduced cellular ATP levels, AMPK induces energy-generating catabolic pathways, such as autophagy, and inhibits energy-consuming anabolic pathways, such as fatty acid synthesis, gluconeogenesis, and protein synthesis, by suppressing mammalian target of rapamycin (mTOR) complex 1 (mTORC1)^8^. Our study also focuses on eukaryotic elongation factor 2 (eEF2), because it lies downstream of AMPK-mTORC1^9^. eEF2 is a GTPase that translocates peptidyl-tRNA from the A-site to P-site of the ribosome, thereby controlling translation elongation. Upon phosphorylation by eEF2 kinase (eEF2K) at Thr56, eEF2 is unable to bind a ribosome and is thus inactivated^10^. eEF2K activity is regulated either negatively or positively by multiple kinases targeting different phosphorylation sites. For example, p70S6 kinase, a substrate of mTORC1, phosphorylates eEF2K Ser366 and inhibits its activity^11^. In contrast, AMPK phosphorylates eEF2K at Ser398^12^, and Ser491/492^13^ and enhances its activity, leading to eEF2 inactivation. Altered AMPK activity^14,15^, mTOR signaling^16^, eEF2 phosphorylation^17,18^, and protein synthesis^17,18^ have been reported in several tissues of several species during hibernation. However, changes of AMPK activity in the brain have been less focused. In squirrels, no change of AMPK activity in the brain was reported^15^. In addition, there has been no systematic analysis of these changes in the brain during hibernation in chipmunks. Considering that eEF2 activity is a major regulator of protein synthesis in neurons^19^, and protein synthesis is a major consumer of cellular energy, we analyzed sequential AMPK-mTORC1-eEF2 signaling and protein synthesis ability in the brain and peripheral tissues of active and hibernating chipmunks.

## Results

### Active and hibernation cycle

**Figure 1:**
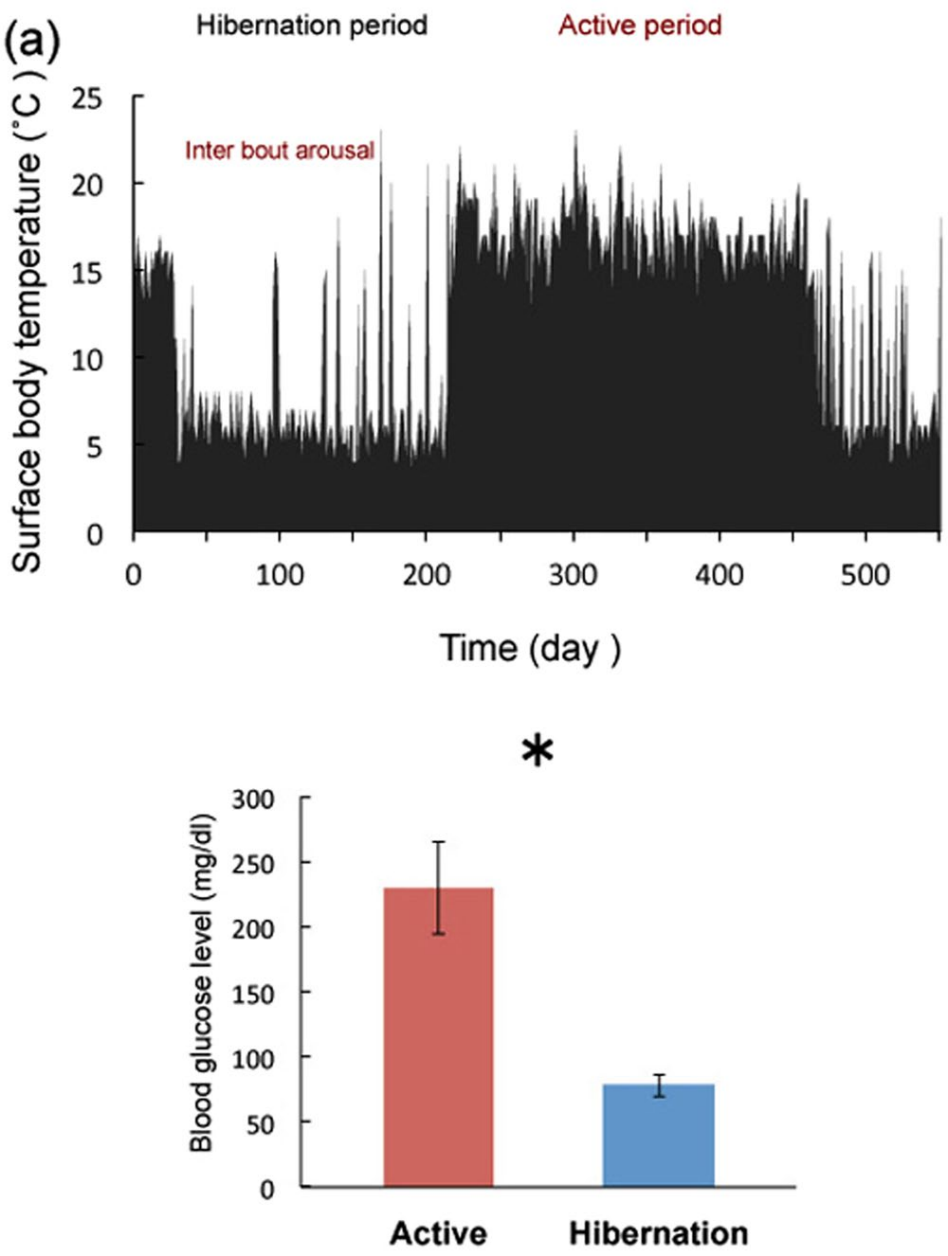
Surface body temperature and blood glucose levels. (**a**) Changes in the surface body temperature as monitored with infrared irradiation thermometer during active and hibernation. The examples of interbout arousals are indicated by arrows. (**b**) Blood glucose levels during each condition. Bars represent mean ± SE (n = 5). *p < 0.05 (Student’s *t*-test).

The surface body temperature of each animal was monitored over a year. Figure 1a shows the typical active and hibernation cycle. The cycle was almost circannual, however, this could be owing to the constant dark condition and low temperature (4 °C), the cycles of some chipmunks displayed free-running (Supplemental Fig. 1).

### Blood glucose level

Tissue samples were collected during active and hibernation periods based on the surface body temperature (Fig. 1a). Interbout arousal periods was avoided for sampling. Glucose is a primary regulator of AMPK; therefore, blood glucose level was measured during both active and hibernation periods (Fig. 1b). As expected, blood glucose was reduced during hibernation compared with the active period. Blood glucose levels were found to be high compared with those in rats or mice (approximately 130 mg/dl). This finding may not be applicable to chipmunks in general and could be attributed to the feeding conditions (*ad libitum*) or genetic traits of the group of chipmunks sampled in this study. Chipmunks are not laboratory animals; our samples were obtained from the wild, therefore, their genetic background may vary. We observed a difference between euthermic and hibernating chipmunks in this study.

### AMPK and acetyl-CoA carboxylase (ACC) phosphorylation

**Figure 2:**
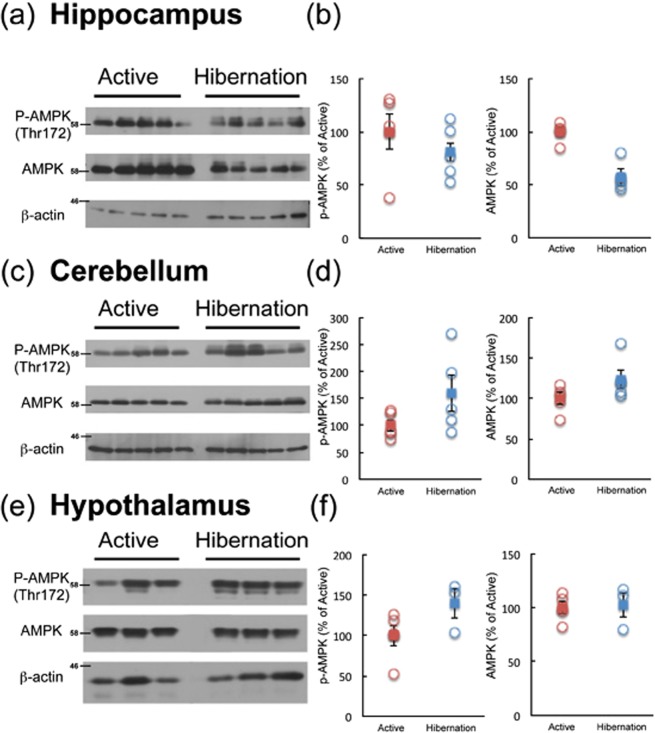
Phosphorylation of AMPKα in the hippocampus (**a**,**b**), cerebellum (**c**,**d**), and hypothalamus (**e**,**f**) of chipmunks during active and hibernation periods. All the samples were applied to the same gel and blotted to a single membrane as displayed. Panels b, d, and f show quantitation of the blots by Image J. Circles represent each band. Squares represent mean ± SE (n = 5 or 3).

**Figure 3:**
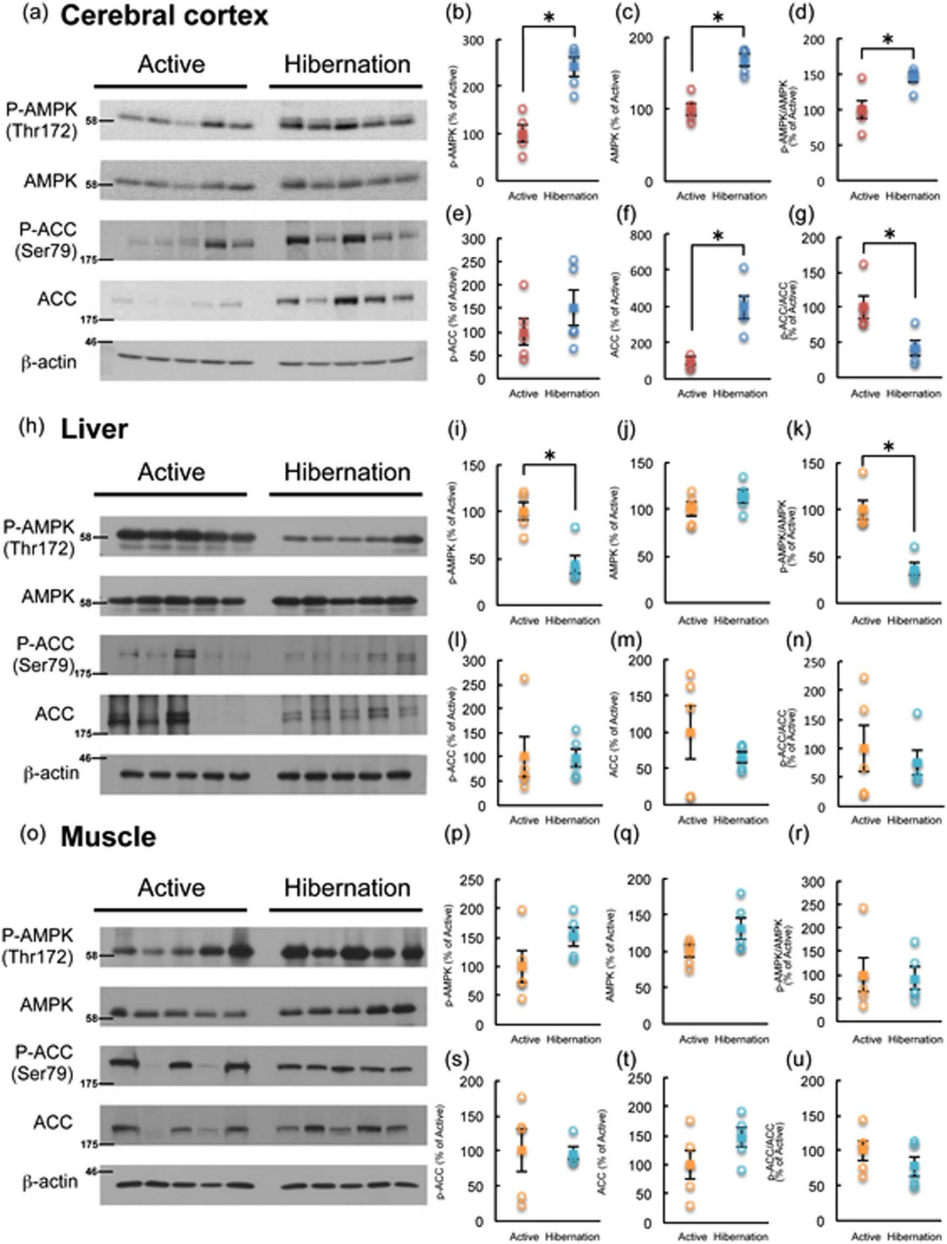
Phosphorylation of AMPKα and ACC in the cerebral cortex (**a**–**g**), liver (**h**–**n**), and skeletal muscle (**o**–**u**) of chipmunks during active and hibernation periods as revealed by Western blotting. All the samples were applied to the same gel and blotted to single membrane as displayed. Each band was quantified by Image J. (**b,i,p**); P-AMPKα, (**c,j,q**); AMPKα, (**d,k,r**); ratio of P-AMPKα/AMPKα, (**e,l,s**); P-ACC, (**f,m,t**); ACC, (**g,n,u**); ratio of P-ACC/ACC. Circles represent each band. Squares represent mean ± SE (n = 5). *p < 0.05 (Wilcoxon rank-sum test (**g,i,k,l,n**) or Student’s *t*-test (all the rest)).

Phosphorylation of AMPKα (Thr172), reflecting its activation, was examined in multiple brain regions of active and hibernating chipmunks by Western blotting. Among the brain regions tested, AMPKα phosphorylation was significantly enhanced during hibernation only in the cerebral cortex, but not in the hippocampus, cerebellum, or hypothalamus (Figs 2, 3). Accordingly, phosphorylation of ACC tended to increase in the cerebral cortex during hibernation compared with the active period (Fig. 3). Because ACC is a direct substrate of AMPK, increase of phospho-ACC indicates the increased net activity of AMPK in the cerebral cortex. In contrast, AMPKα phosphorylation was somewhat reduced in the liver and unchanged in the skeletal muscles of hibernating chipmunks (Fig. 3), despite low blood glucose levels. These results indicate that AMPK is activated only in the cerebral cortex during hibernation.

### Downstream signaling of AMPK

**Figure 4:**
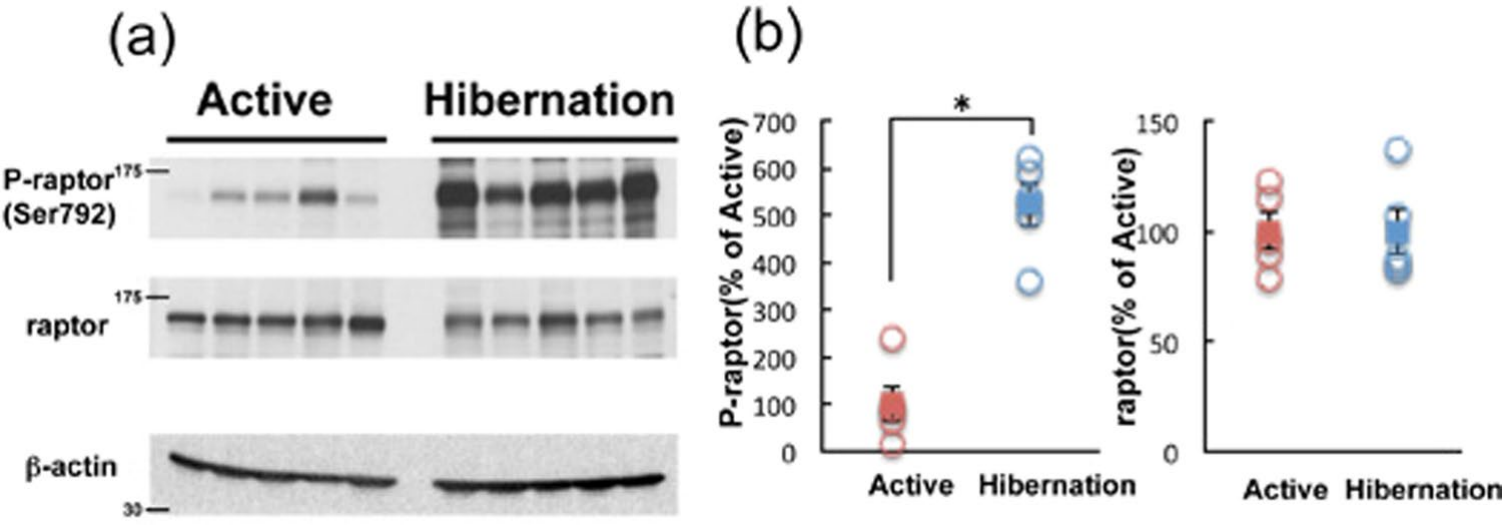
Phosphorylation of raptor in cerebral cortex of chipmunks during active and hibernation periods (**a**,**b**). All the samples were applied to the same gel and blotted to a single membrane as displayed (**a**). Panel b shows quantitation of the blots by Image J. Circles represent each band. Squares represent mean ± SE (n = 5). *p* < 0.05 (Student’s *t*-test).

**Figure 5:**
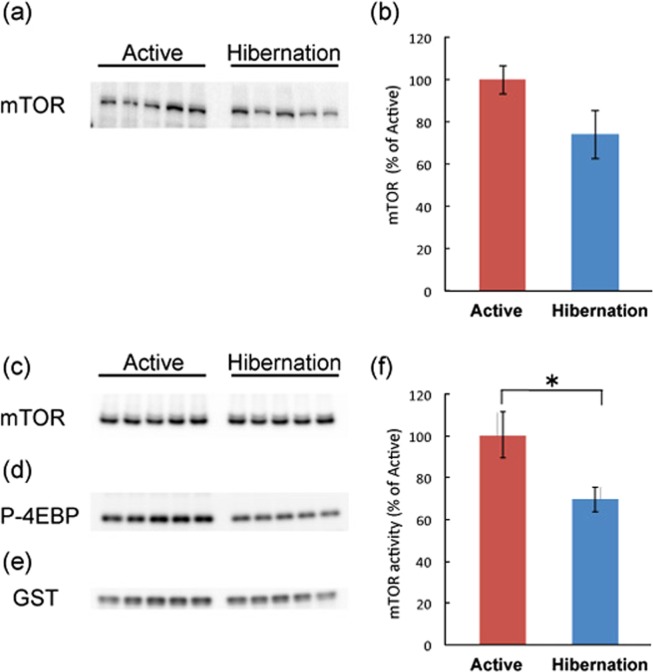
Levels of mTOR in the cerebral cortex of chipmunks during active and hibernation periods. All the samples were applied to the same gel and blotted to single membrane as displayed (**a**). Panel b shows quantitation of the blots by Image J. Bars represent mean ± SE. (n = 5) Kinase activity of mTORC1 during active and hibernation periods is shown in f. Panels c shows immunoprecipitated mTOR. Panel d shows phosphorylated GST-4EBP after *in vitro* phosphorylation. Panel e shows GST, as the control of total GST-4EBP in the reaction mixture. All the samples were applied to the same gel and blotted to single membrane as displayed (**c**–**e**). Kinase activity (**f**) is calculated by (GST-P-4EBP (**d**)/GST (**e**))/mTOR (**c**). Bars represent mean ± SE (n = 5). *p < 0.05 (Student’s *t*-test).

We then examined the signaling status of mTORC1, a major downstream target of AMPK, in the cerebral cortex of active and hibernating chipmunks. Raptor is an indispensable component of mTORC1^20,21^ and is phosphorylated at Ser792 by AMPK^22^, which suppresses mTORC1 activity. Phosphorylation of raptor was increased in the cerebral cortex of hibernating chipmunks (Fig. 4), suggesting reduced activity of mTORC1; therefore, mTORC1 kinase activity was directly measured. Therefore, equal amounts of mTOR were immunoprecipitated (Fig. 5c) from cerebral cortical tissues of each period and incubated with recombinant GST-4EBP, as a substrate, with ATP, followed by Western blotting with an anti-phosph-4EBP(Thr37/46) (Fig. 5d). As shown in Fig. 5f, mTORC1 activity was downregulated in the cerebral cortex of hibernating chipmunks. In addition, total mTOR protein levels are lower in the cortex of hibernating chipmunks, although it is not significant (Fig. 5a,b). Cumulatively, net mTORC1 activity in the cortex of hibernating chipmunks is lower than that in the cortex of active animals.

**Figure 6:**
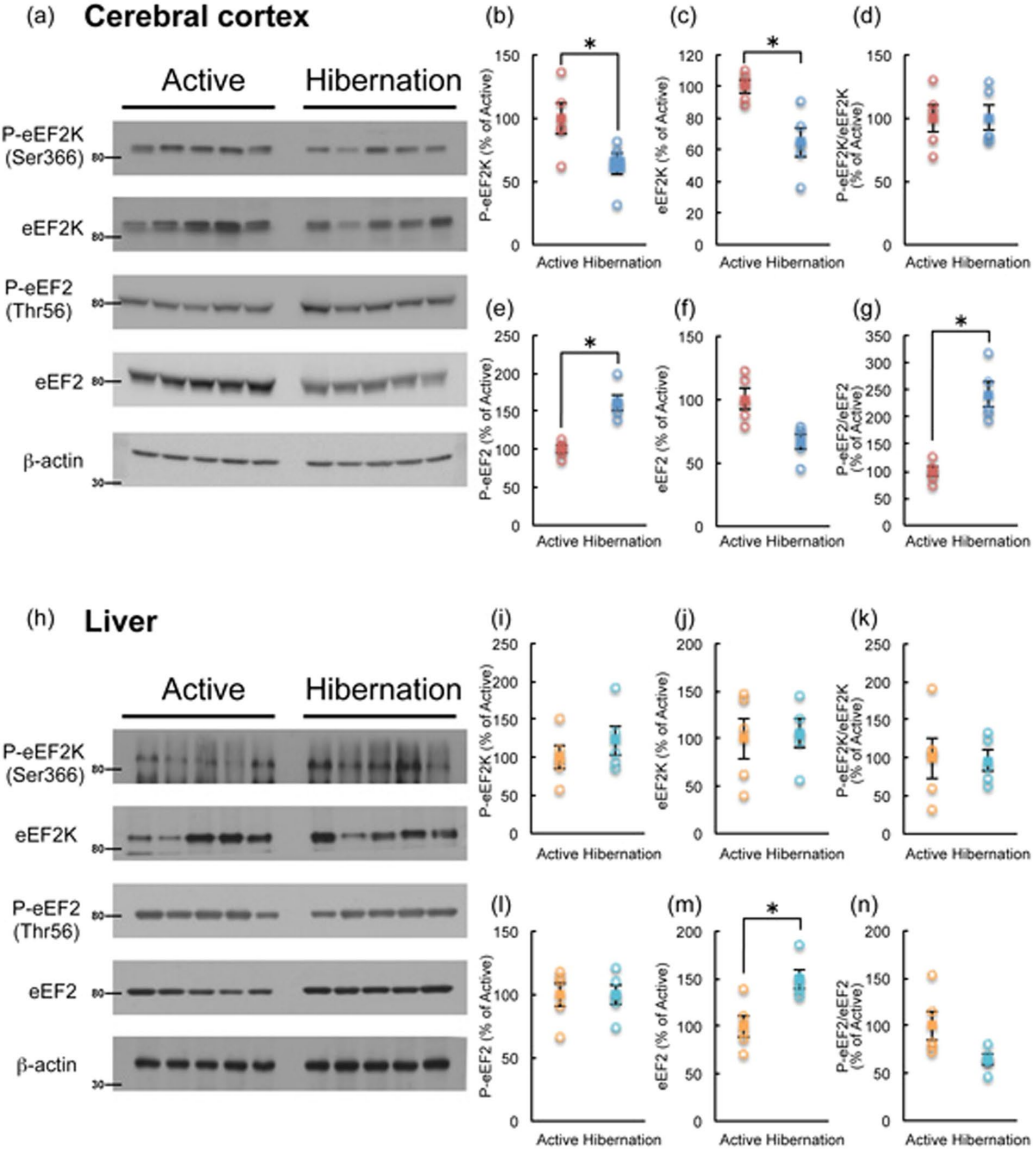
Phosphorylation of eEF2K and eEF2 in the cerebral cortex (**a**–**g**) and liver (**h**–**n**) of chipmunks during active and hibernation periods. All samples were applied to the same gel and blotted to a single membrane as displayed. Each band was quantified by Image J. (**b**,**i**); P-eEF2K, (**c**,**j**); eEF2K, (**d**,**k**); ratio of P-eEF2K/eEF2K, (**e**,**l**); P-eEF2, (**f**,**m**); eEF2, (**g,n**); ratio of P-eEF2/eEF2. Each circle represents the each band. Squares represent mean ± SE (n = 5) *p < 0.05 (Student’s *t*-test).

eEF2 activity is suppressed by eEF2K-mediated phosphorylation at Thr56, which in turn reduces translation elongation efficiency^10^. eEF2K activity is downregulated by phosphorylation at Ser366 by mTORC1 signaling; therefore, reduced eEF2K phosphorylation at Ser366 would allow eEF2 phosphorylation (inactivation) and thus reduce protein synthesis^11^. eEF2K phosphorylation at Ser366 was decreased in the cerebral cortex of hibernating chipmunks (Fig. 6a,b). This finding is consistent with the reduced mTORC1 activity and suggests increased activity of eEF2K, in line with the upregulation of eEF2 phosphorylation at Thr56 (Fig. 6a,e). Conversely, these changes were not observed in the liver (Fig. 6h,i,l). The results suggest that translation elongation and ensuing total protein synthesis are downregulated in the cerebral cortex, but not in the liver, of hibernating chipmunks.

### Rate of protein synthesis in active and hibernating tissues

**Figure 7:**
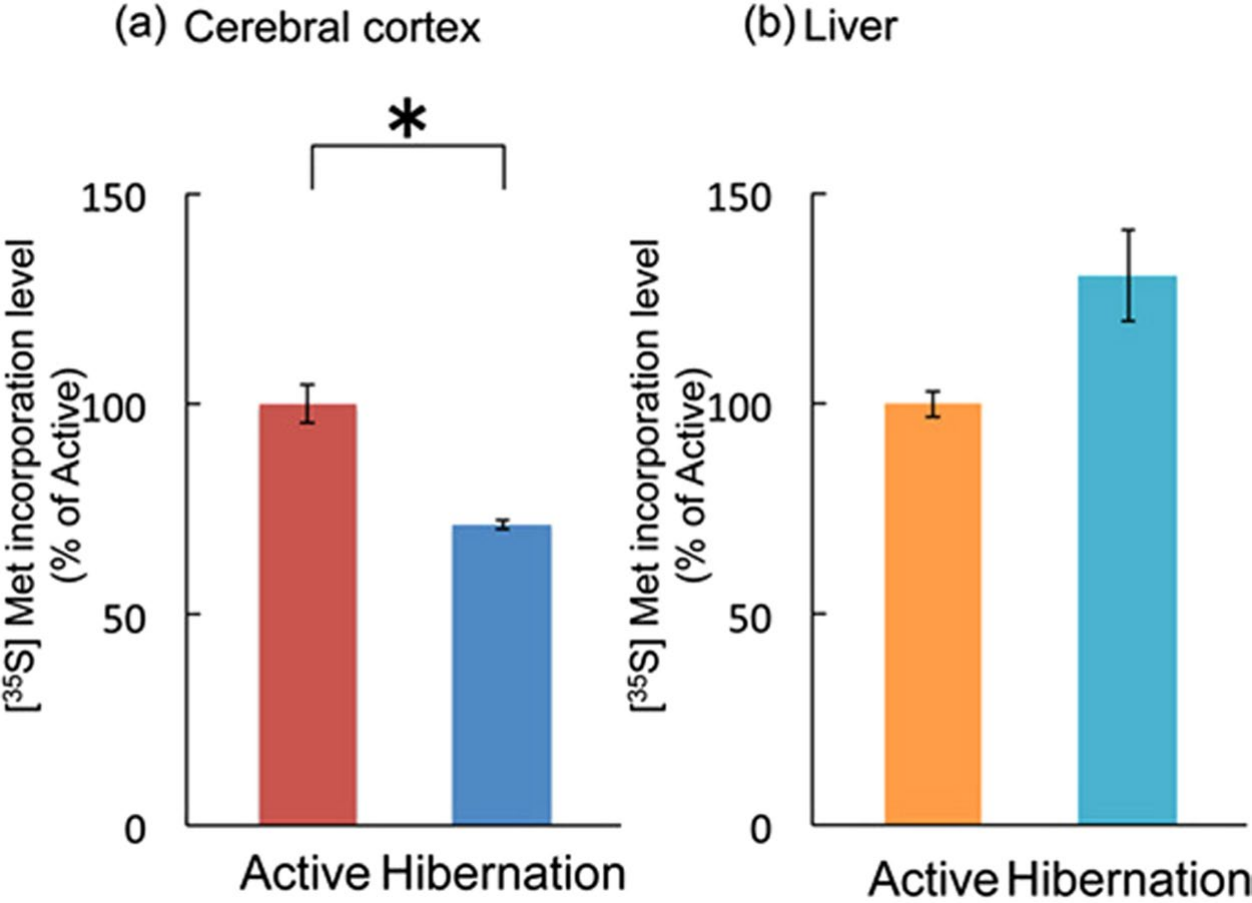
Comparison of protein synthesis capacity *in vitro* as measured by [^35^S]methionine incorporation using chipmunks between cerebral cortex (**a**) and liver (**b**) during active and hibernation periods. Bars represent mean ± SD (n = 5). *p < 0.05 (Student’s *t*-test).

Protein synthesis capacity was examined in the cerebral cortical and liver tissues during the active and hibernation periods by *in vitro* [^35^S]methionine incorporation as previously reported^19^. In accordance with the decrease in mTORC1 activity and inactivation of eEF2 (phosphorylation), protein synthesis rate was lower in the cerebral cortex of hibernating chipmunks than in that of active chipmunks, whereas liver protein synthesis rate did not differ between the active and hibernation periods (Fig. 7).

## Discussion

In this study, we analyzed the signaling pathways underlying the altered metabolic and protein synthesis rates in the cerebral cortex of hibernating chipmunks. During hibernation, the cerebral cortex exhibited (1) an increase of AMPKα phosphorylation (indicating elevated AMPK activity), (2) suppression of mTORC1 activity accompanied with raptor phosphorylation, (3) phosphorylation (inactivation) of eEF2, resulting in (4) reduced ability of protein synthesis as evidenced by lower *in vitro* [35 S]methionine incorporation.

Among the brain regions tested, significant AMPK activation was observed only in the cerebral cortex of hibernating chipmunks. However, no changes in the liver and skeletal muscle were observed during either active or hibernation period. The lack of increase in AMPK in the liver and skeletal muscles is in accordance with previous results from studies of the ground squirrel^15^. Liver and skeletal muscles synthesize and store glycogen; therefore, AMPK activity may not quickly respond to falling blood glucose levels. Instead, AMPK activity may change depending on the period of torpor and fasting. Indeed, AMPKα phosphorylation in the liver and skeletal muscles of rats is not altered in a short-term fasting^23^. Further, long-term caloric restriction rather decreased phospho-AMPKα levels in rat liver^24^. In contrast to peripheral tissues, phosphorylation status of AMPKα and ACC was increased in the cortex of hibernating chipmunks, a result not observed in the whole brain of thirteen striped-ground squirrels^15^. The increase in AMPK activity observed in the cerebral cortex of ground squirrels may have been obscured by a lack of change in the rest of the brain. In the hypothalamus of golden mantled ground squirrels, phosphorylation of ACC, but not AMPKα, was increased^14^, which is in partial accordance with our finding of unaltered AMPKα phosphorylation in the hypothalamus of chipmunks. In the brain, glycogen stores and fatty acid beta-oxidation are limited and almost all energy is derived from blood glucose. Therefore, the reduction in blood glucose levels may rapidly affect AMPK activity in the cerebral cortex, but not in energy-storing peripheral organs. However, this does not explain why phosphorylation of AMPKα in other brain regions was unaltered. Further studies are required to reveal possible differences in the regulation of AMPKα phosphorylation status and its activity among brain regions.

Along with AMPK activation, eEF2 phosphorylation (inactivation) was observed in the cerebral cortex, but not in the liver. AMPK is known to phosphorylate eEF2K directly^12,13^ and to activate indirectly through the suppression of the mTORC1 pathway^11^. In this study, both total and phosphorylated eEF2K at Ser366 were reduced in the cortex of hibernating chipmunks. Thus, direct phosphorylation of eEF2K by AMPK may contribute to the activation of eEF2K, thus inhibiting eEF2 activity.

Hallenbeck’s group has reported eEF2 phosphorylation, prolonged ribosomal transit time that indicates slowing elongation rate, and reduced protein synthesis in the brains of hibernating ground squirrels^17,18^. Therefore, eEF2 inactivation in the brain may be a common mechanisms for reducing protein synthesis in hibernating rodents. It has also been reported the Decreased activity of protein phosphatase 2A, the dephosphorylation enzyme for eEF2 has also been reported in the brain of hibernating ground squirrels^18^. It may also occur in thecerebral cortex of hibernating chipmunks.

In addition to the cellular energy status, cell type-specific Ca^2+^ dynamics may influence AMPKα and eEF2 phosphorylation. AMPKα is phosphorylated by calcium-calmodulin-dependent protein kinase kinase (CaMKK)^25–27^ and eEF2 is phosphorylated by eEF2K, (also known as calcium/calmodulin dependent protein kinase III)^28^. The activity of both kinases is Ca^2+^-dependent. Intracellular Ca^2+^ dynamics are known to be altered in cardiac muscle cells during hibernation^29^, suggesting that cell type-specific changes in Ca^2+^ mobilization lead to differential activity of AMPK and eEF2 among tissues. Indeed, cold-stress-induced eEF2K activation via Ca^2+^ upregulation was previously reported^30^.

Another possible explanation for the cerebral cortex-specific activation of AMPK is the direct involvement of the hibernation protein complex (HPc) comprising HP20, HP25, and HP27^2^. These HPs have collagen-like domains in the N-terminal region and form a triple-helix^31^. Structurally, HPc is predicted to belong to the C1q protein family^31,32^. Adiponectin and CTRPs, other members of this family, are reported to induce AMPK activation^33,34^. Because HPc is suggested to act in the brain^1^, HPc in the cortex may directly activate AMPK.

Although the phosphorylation levels of the molecules tested were enhanced in the cortex during hibernation, this is not a general phenomenon (for instance, due to massive loss of phosphatase activity). In fact, phosphorylation of Akt was decreased in the cerebral cortex (Supplemental Fig. 2), possibly due to the downregulation of mTORC2 and/or low levels of neural activity.

As previously reported^18^, protein synthesis ability in the cortical tissue is downregulated as revealed by *in vitro* assay at 37 °C. This finding reflects the status of translation machinery, in other words, “readiness for protein synthesis”. This suggests that the function of translation machinery in the hibernating cortex is suppressed. Under colder condition during hibernation, actual protein synthesis *in vivo* may be further downregulated. In fact, protein synthesis as revealed by [^14^C]leucine incorporation was markedly decreased in the liver and heart, (in addition to the brain) of hibernating squirrels *in situ* at 7 °C body temperature^18^.

The remaining unsolved question regards the relationship between circannual (hibernation) and circadian rhythms. The changes in each signaling molecule and its phosphorylation status may have been modified by circadian rhythms because the animals in the current study were housed in constant darkness and thus their rhythms may have free-run. Indeed, circadian change in AMPK activity has been previously reported in rats^35^. Unfortunately, we have no data in this study; however it is an important point in hibernation studies.

Based on the present study, we suggest that the increased activity of AMPK and decreased protein synthesis may contribute to the low-temperature resistance of brain cells during hibernation. Future studies are required to assess the involvement of hibernation proteins in these metabolic changes and low-temperature resistance.

## Materials and Methods

### Materials

Anti-AMPKα, anti-phospho-AMPKα (Thr172), anti-ACC, anti-phospho-ACC (Ser79), anti-eEF2K, anti-phospho-eEF2K(Ser366), anti-Akt, anti-phospho-Akt(Ser473), anti-p44/42MAPK, and anti-phosopho-p44/42MAPK (Thr202/Tyr204) were purchased from Cell Signaling Technology. Anti-mTOR was obtained from Immuno-Biological Laboratories. Anti-eEF2 and anti-phospho-eEF2 antibodies were prepared as described previously^36^. Validation of antibody specificity to chipmunk proteins is shown in Supplemental Fig. 4. [^35^S]methionine and Protein G sepharose were purchased from Perkin Elmer and GE Healthcare, respectively.

### Antibody validation

The target molecules analyzed in this study are well conserved among species, and tge amino acid sequences and phosphorylation sites of AMPKα1 in mouse, rat, rabbit, human and squirrel are shown in Supplemental Fig. 3. To assess the specificity of the antibodies (anti-AMPKα, anti-P-AMPKα, anti-eEF2 and anti-P-eEF2), freshly prepared brain lysate was applied to SDS-PAGE and transferred and blotted in full size (from top to bottom). Antigen absorption was also performed. cDNAs of rat AMPKα^37,38^ fused with GST and rat eEF2^19^ tagged with Flag were transfected to HEK293T cells and starved with serum to increase phosphorylated forms. GST-AMPKα and Flag eEF2 were pull-down with Glutathione-sepharose and anti-DYKDDDDK(Flag)-agarose, respectively. Western blots of the antigens were shown in Supplemental Fig. 4. Antigens bound to resins were incubated with each antibody. As shown in Supplemental Fig. 4, each antibody recognized a single band of similar molecular weight (rat or human AMPKα and eEF2 both total and phosphorylated forms). Signals were completely abolished when using antigen-absorbed antibodies (Supplemental Fig. 4).

### Animals

All the animal experiments were conducted in compliance with the protocol which was reviewed by the Institutional Animal Care and Use Committee and approved by the President of Niigata University (Permit Number: Niigata Univ. Res.258-1). Protocols were performed in accordance with the Guiding Principles for Care and Use of Laboratory Animals (NIH, USA). Male chipmunks (*Tamias sibiricus*) less than one year of age were purchased from Arcland Sakamoto Co. Ltd. The chipmunks were housed individually at 23 °C under 12h-light/dark cycle conditions and provided a standard rat diet and water *ad libitum*. The animals were maintained for at least six months to acclimate to laboratory conditions. After checking general health conditions, they were transferred to the experimental condition of 4 °C and constant darkness. Periodical changes in the body temperature were monitored with an infrared irradiation thermometer once a day more than one year. Only individuals that exhibited periodical changes (Fig. 1a as an example) were used for the study. There are certain populations of chipmunks that do not exhibit temperature change and these individuals were excluded from this study. The onset, duration and termination of hibernation were recognized by monitoring the surface body temperature^1^. Animals (five chipmunks of each group (active: surface body temperature >18 °C and hibernation: surface body temperature <6 °C) were deeply anesthetized with carbon dioxide and sacrificed by decapitation and tissues were collected at 13:00–15:00 h from October to November. Tissue and blood samples were collected, immediately frozen in liquid nitrogen, and stored at −80 °C until further analysis.

### Measurement of blood glucose level

The blood glucose levels during each period were measured using self-measurement instrument (Freestyle freedom, Nipro). After thawing the cryopreserved samples on ice, 0.3 μl blood was used for measuring the blood glucose levels.

### Tissue extracts, electrophoresis, and Western blotting

Tissue extracts, electrophoresis, and Western blotting were performed as previously reported^19^. Briefly, frozen tissue samples were weighted and homogenized in ten volumes of lysis buffer (62.5 mM Tris-HCl (pH6.8), 2% SDS, Complete protease inhibitor cocktail(Roche Applied Science Ltd.), and PhosStop(Roche Applied Science Ltd.)). After centrifugation (15,000 rpm × 60 min), supernatants were collected. The protein concentration of each sample was determined by Micro BCA (PIERCE Ltd.). Equal amounts of protein (50 μg/lane for indicated molecules and 10 μg/lane for β-actin) were subjected to sodium dodecyl sulfate–polyacrylamide gel electrophoresis and transferred to plolyvinylidene fluoride membranes. The membranes were cropped around the appropriate molecular size to save the amount of antibodies. The membranes were blocked in TNT (150 mM NaCl, 10 nM Tris-HCl (pH 7.4), and 0.05% tween-20) containing 10% BSA and then incubated with the indicated primary antibody overnight. After washing with TNT, blotted membranes were incubated with horseradish-peroxidase (HRP)-conjugated anti-rabbit IgG (1:10000 dilution; Dako cytomation) or HRP-conjugated anti-mouse IgG (1:10000 dilution; Jackson immune research Inc.) for 1 h. After washing, peroxidase activity was detected by chemiluminescence reagents (Western Lightning, PerkinElmer Life Science) and visualized on X-ray film (Fujifilm Medical Co., Ltd). The quantity of protein expressed was quantified by “Image J” software for Macintosh OS X and standardized by β-actin. β-actin blot was performed to validate the protein concentration. It is impossible to reprobe with anti-β-actin in the same blots, for example AMPK because the amount of total applied protein is too much for β-actin blot. The actin signal is saturated and is not applicable for quantification. If the protein amount is adjusted to actin level, other molecules cannot be detected. All blots presented in this study are from the same samples so that actin blots are the same in all the Western blot experiments. In some cases, brightness and/or intensity of the gels were manipulated to clarify the signals. The manipulation covered the entire area of the blots to ensure that the changes were made equally to each band.

### mTOR immunoprecipitation and *in vitro* kinase assay

The kinase activity of mTOR in tissue samples was measured as in previous reports^39,40^. Tissue samples were homogenized in 20 volumes of lysis buffer (50 mM Tris–HCl, pH 8.0, 150 mM, NaCl, 1 mM EDTA, 5 mM EGTA, 20 mM glycerophosphate, 1 mM dithiothreitol, 1 mM protease inhibitor cocktail (Complete, Roche), phosphatase inhibitor cocktail (PhosStop, Roche)) and centrifuged for 30 min at 4 °C. The protein concentration of the supernatant was determined and 800 μg of each sample was used for immunoprecipitation. Samples were pre-absorbed with Protein G Sepharose for 60 min at 4 °C, centrifuged for 3 min at 4 °C, then mixed with mouse anti-mTOR antibody (1 μg) and incubated overnight at 4 °C. To measure kinase activity more accurately, saturation amount of lysates and a small amount of antibody was used to precipitate same amount of mTOR from active and hibernating chipmunks. Protein G sepharose was added to the samples and incubated for 2 h at 4 °C. After washing, immunoprecipitates were mixed with kination buffer including recombinant GST 4E-BP and ATP, and incubated for 30 min at 30 °C. The reaction was stopped by adding SDS sample buffer and boiling for 5 min at 95 °C. In the same membrane, mTOR and P-4EBP was immunoblotted. After that, membrane was reprobed with anti-GST antibody. mTOR kinase activity was calculated by the signal intensity of (P-4EBP/GST)/mTOR.

### Metabolic labeling by [^35^S]methionine

Protein synthesis was measured by [^35^S]methionine incorporation^19^. Samples of brain and liver were gently homogenized with 10 volumes of DMEM and protein concentration was determined. Each sample (50 μg) was incubated with 10 µCi of [^35^S]methionine for 30 min at 37 °C. Samples were then lysed in equal volumes of 1 M NaOH containing casein as carrier, and incubated for 30 min at 42 °C. An equal volume of 20% ice-cold trichloroacetic acid was added to each sample, and the mixture was incubated for 1 h at 4 °C. After centrifugation, free methionine and methionine incorporated into protein were estimated by determining the radioactivity of supernatants and the pellet, respectively. Protein synthesis was estimated based on the ratio of methionine in the pellet to total methionine.

### Statistical analysis

All values are presented as mean ± SE. Differences between groups were statistically determined by Student’s t-test when data were normally distributed or Wilcoxon rank-sum test when data were not normally distributed. Analysis were performed by the statistical software “R” (version 3.1.2). p < 0.05 were considered statistically significant.

## Supplementary information

Supplemental Figures

## Data Availability

All data generated or analyzed during this study are included in this published article (and its Supplementary Information files).
