# Supplementary material for: AMPK activation, eEF2 inactivation, and reduced protein synthesis in the cerebral cortex of hibernating chipmunks
Source: Sci Rep. 2019 Aug 15;9:11904. doi: 10.1038/s41598-019-48172-7 (PMC6695389; doi:10.1038/s41598-019-48172-7)
Supplement: Supplementary file 1 — Supplemental Figures [file 41598_2019_48172_MOESM1_ESM.pdf]

Supplemental Figures

AMPK activation, eEF2 inactivation, and reduced protein synthesis in the cerebral cortex of hibernating chipmunks

Shintaro Yamada, Taito Kamata, Hiroyuki Nawa, Tsuneo Sekijima and Nobuyuki Takei

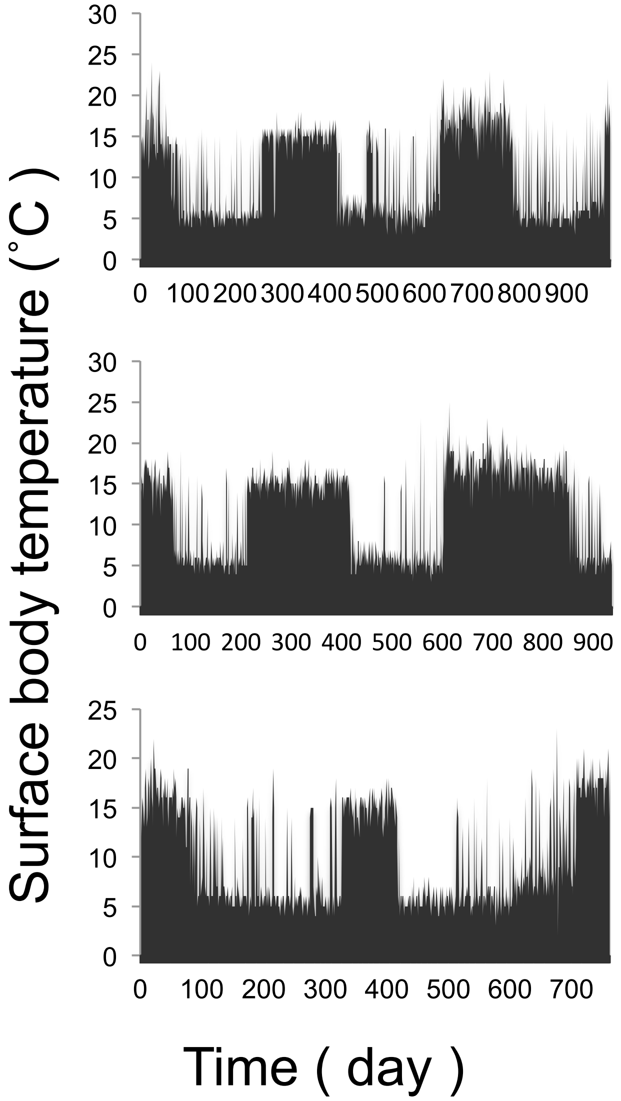

Supplemental Figure 1

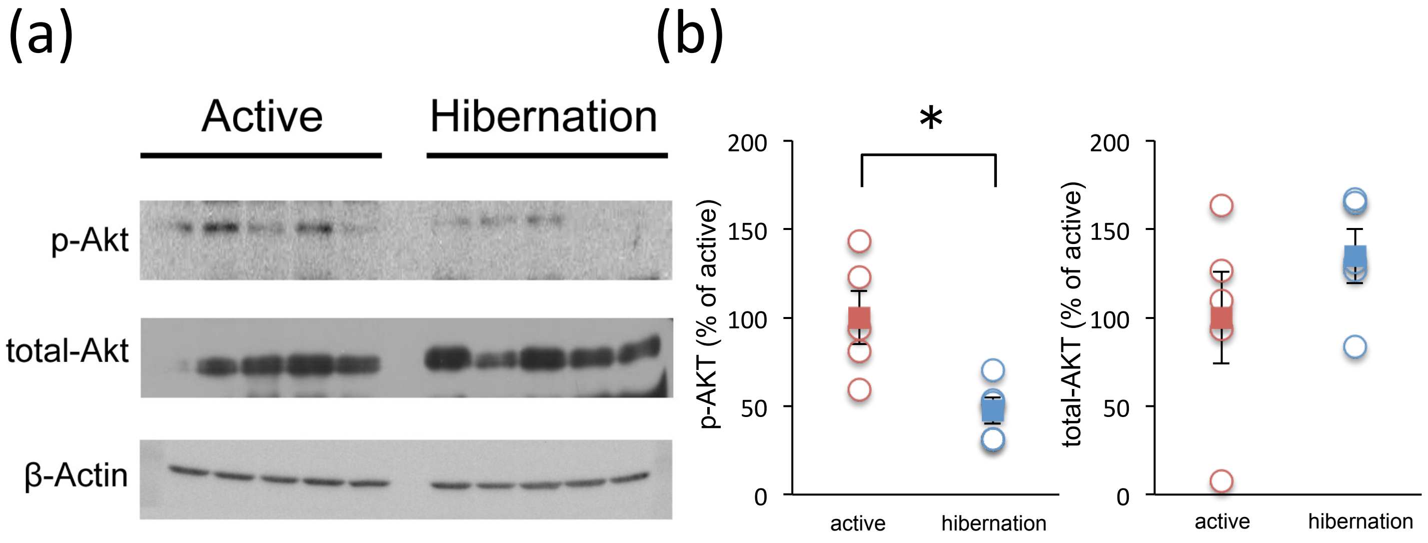

Supplemental Figure. 2

```
              ....|....| ....|....| ....|....| ....|....| ....|....| ....|....|
                  5         15         25         35         45         55
M.musc        IGHYILGDTL GVGTFGKVKV GKHELTGHKV AVKILNRQKI RSLDVVGKIR REIQNLKLFR
R.norv        IGHYILGDTL GVGTFGKVKV GKHELTGHKV AVKILNRQKI RSLDVVGKIR REIQNLKLFR
O.cuni        IGHYILGDTL GVGTFGKVKV GKHELTGHKV AVKILNRQKI RSLDVVGKIR REIQNLKLFR
H.sapi        IGHYILGDTL GVGTFGKVKV GKHELTGHKV AVKILNRQKI RSLDVVGKIR REIQNLKLFR
U.parr        IGHYILGDTL GVGTFGKVKV GKHELTGHKV AVKILNRQKI RSLDVVGKIR REIQNLKLFR
I.trid        ---------- ---------- ---------- ---------- ---------- ----------

              ....|....| ....|....| ....|....| ....|....| ....|....| ....|....|
                  65         75         85         95        105        115
M.musc        HPHIIKLYQV ISTPSDIFMV MEYVSGGELF DYICKNGR-- ---------- ---LDEKESR
R.norv        HPHIIKLYQV ISTPSDIFMV MEYVSGGELF DYICKNGR-- ---------- ---LDEKESR
O.cuni        HPHIIKLYQV ISTPSDIFMV MEYVSGGELF DYICKNGR-- ---------- ---LDEKESR
H.sapi        HPHIIKLYQV ISTPSDIFMV MEYVSGGELF DYICKNGR-- ---------- ---LDEKESR
U.parr        HPHIIKLYQV ISTPSDIFMV MEYVSGGELF DYICKNGR-- ---------- ---LDEKESR
I.trid        ---------- --------MV MEYVSGGELF DYICKNGR-- ---------- ---LDEKESR

              ....|....| ....|....| ....|....| ....|....| ....|....| ....|....|
                 125        135        145        155        165        175
M.musc        RLFQQILSGV DYCHRHMVVH RDLKPENVLL DAHMNAKIAD FGLSNMMSDG EFLRTSCGSP
R.norv        RLFQQILSGV DYCHRHMVVH RDLKPENVLL DAHMNAKIAD FGLSNMMSDG EFLRTSCGSP
O.cuni        RLFQQILSGV DYCHRHMVVH RDLKPENVLL DAHMNAKIAD FGLSNMMSDG EFLRTSCGSP
H.sapi        RLFQQILSGV DYCHRHMVVH RDLKPENVLL DAHMNAKIAD FGLSNMMSDG EFLRTSCGSP
U.parr        RLFQQILSGV DYCHRHMVVH RDLKPENVLL DAHMNAKIAD FGLSNMMSDG EFLRTSCGSP
I.trid        RLFQQILSGV DYCHRHMVVH RDLKPENVLL DAHMNAKIAD FGLSNMMSDG EFLRTSCGSP

              ....|....| ....|....| ....|....| ....|....| ....|....| ....|....|
                 185        195        205        215        225        235
M.musc        NYAAPEVISG RLYAGPEVDI WSSGVILYAL LCGTLPFDDD HVPTLFKKIC DGIFYTPQYL
R.norv        NYAAPEVISG RLYAGPEVDI WSSGVILYAL LCGTLPFDDD HVPTLFKKIC DGIFYTPQYL
O.cuni        NYAAPEVISG RLYAGPEVDI WSSGVILYAL LCGTLPFDDD HVPTLFKKIC DGIFYTPQYL
H.sapi        NYAAPEVISG RLYAGPEVDI WSSGVILYAL LCGTLPFDDD HVPTLFKKIC DGIFYTPQYL
U.parr        NYAAPEVISG RLYAGPEVDI WSSGVILYAL LCGTLPFDDD HVPTLFKKIC DGIFYTPQYL
I.trid        NYAAPEVISG RLYAGPEVDI WSSGVILYAL LCGTLPFDDD HVPTLFKKIC DGIFYTPQYL

              ....|....| ....|....| ....|....| .
                 245        255        265
M.musc        NPSVISLLKH MLQVDPMKRA AIKDIREHEW F
R.norv        NPSVISLLKH MLQVDPMKRA TIKDIREHEW F
O.cuni        NSSVISLLKH MLQVDPMKRA TIKDIREHEW F
H.sapi        NPSVISLLKH MLQVDPMKRA TIKDIREHEW F
```

Supplemental Figure 3

Legends to Supplemental Figures

Supplemental Figure 1. Examples of active-hibernation rhythms of surface body temperature of chipmunks. Note some of the individuals looks free-running of rhythm over a year.

Supplemental Figure 2. Phosphorylation of Akt in cerebral cortex of chipmunks during active and hibernation periods. Panel b shows quantitation of the blots by Image J. Each circle represents the each band. Squares represent mean±SE (n=5). *p<0.05 (Student *t*-test)

Supplemental Figure 3. Comparison of amino acid sequences of different species. M. musc: mouse, R. norv: rat, O. cuni: rabbit, H. sapi: human, U. parr: arctic ground squirrel, I. trid: thirteen-lined ground squirrel. Red "T" indicates phosphorylation amino acid residue.

Supplemental Figure 4. Recombinant antigens were prepared as described in materials& methods. Rat recombinant GST-AMPKα (a, b) and Flag-eEF2 (c, d) were applied to SDS-PAGE and transferred to the PVDF membrane. Western blotting was performed using anti-total AMPKα (a), anti-P-AMPKα (b), anti-total eEF2 (c) and anti-P-eEF2 (d). Purified antigens were used for absorption of each antibody. Antibody specificity was checked as follows. Same amount (40 μg) of the lysates of newly sacrificed chipmunk brain were applied to Western blotting using anti-AMPKα (e, f), anti-P-AMPKα (g, h) , anti-total eEF2 (i, j) and anti-P-eEF2 (k,l) antibodies. f, h, j, l were blotted with antigen-absorpted antibodies and images were obtained in longer exposure time.

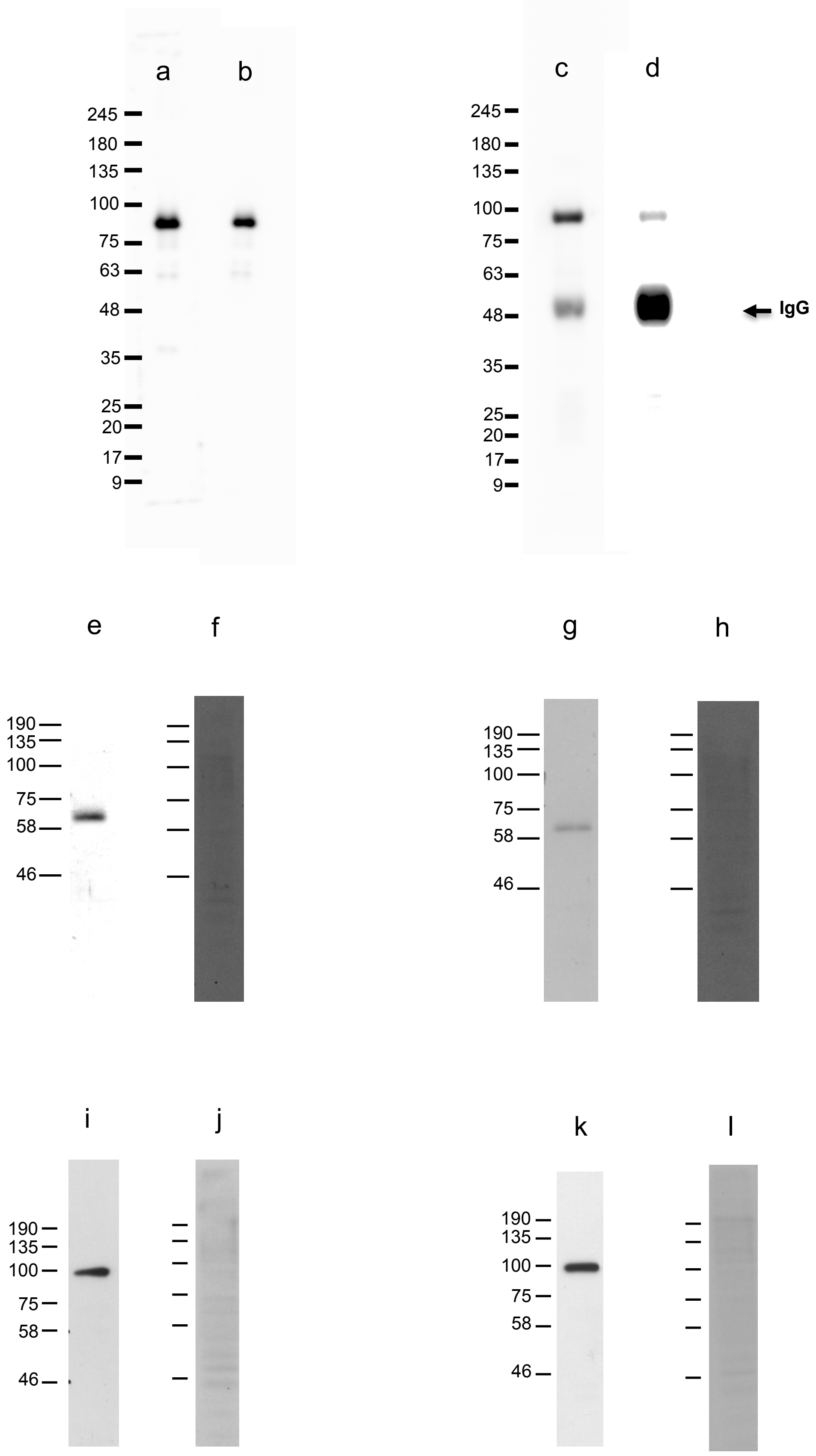

Supplemental Figure 4
